# 14-3-3 Eta protein as a novel biomarker in early detection of uveitis in Egyptian juvenile idiopathic arthritis and rheumatoid arthritis patients: Diagnostic and prognostic value

**DOI:** 10.1515/rir-2024-0030

**Published:** 2025-01-09

**Authors:** Dalia Salah Saif, Manar Fawzy Dawoud, Abeer Medhat, Dina Rifaat Al Sharaki, Dina Salem Fotoh

**Affiliations:** Rheumatology, Rehabilitation, and Physical medicine department, Faculty of Medicine, Menoufia University, Shibin El Kom Egypt; Ophthalmology Department, Faculty of Medicine, Menoufia University, Shibin El Kom Egypt; Clinical pathology Department, Faculty of Medicine, Menoufia University, Shibin El Kom Egypt

**Keywords:** rheumatoid arthritis, juvenile idiopathic arthritis, 14-3-3 Eta protein, disease activity score, health assessment questionnaire

## Abstract

**Background and Objectives:**

Juvenile Idiopathic Arthritis (JIA) and Rheumatoid arthritis (RA) are autoimmune chronic inflammatory disorders of undetermined cause. Uveitis is one of the commonest and most dangerous extra-articular manifestations of JIA and RA presenting chronic anterior uveitis with non-specific biomarkers for its early detection. We evaluated the role of serum 14-3-3 Eta protein to assess its potential role as a novel biomarker for the early detection of uveitis in Egyptian JIA and RA patients as well as its correlation with disease activity.

**Methods:**

A case-control study included three patient groups: group I includes 42 JIA patients, group II includes 42 RA patients, and an equal number of apparently healthy individuals matched in sex and age for each group of patients as controls, recruited from the rheumatology outpatient clinic. All participants were subjected to clinical examination, laboratory investigations with assessment of serum levels of 14-3-3 Eta protein, and ophthalmologic investigations to assess disease activity, eye affection, and its relation to 14-3-3 Eta protein level, and other disease variables among those patients.

**Results:**

a statistically significant difference was estimated between the two patients’ groups and controls regarding 14-3-3 Eta protein level. 14-3-3 Eta protein has a significant positive correlation with disease activity in JIA and RA patients. Also, RA patients with clinical uveitis had higher levels of the 14-3-3 Eta protein, while there were no significant differences among JIA patients with or without uveitis.

**Conclusion:**

14-3-3 Eta protein is a potential diagnostic biomarker in early detection of uveitis in RA patients, as it is higher in patients versus controls especially those with uveitis with a cut-off point 57.5, at which patients must have a thorough eye examination to receive early intervention and, to prevent complications, while it doesn’*t* have the same role in JIA patients. 14-3-3 Eta protein is a potential diagnostic and prognostic marker for JIA and RA being correlated with disease activity.

## Introduction

Juvenile Idiopathic Arthritis (JIA) is considered the most common heterogeneous group of arthritis of unknown etiology beginning in the pediatric population before sixteen years of age and lasting for more than six weeks.^[1]^ JIA is a major cause of acquired impairment and significant handicapping in children and adolescents because of aggressive joint involvement in adult life in many JIA patients. About 1–3 per 1000 children are affected by JIA worldwide.^[1]^

Up till now, JIA diagnosis has been difficult as being represented with inflammatory arthritis with no specific accurate test to confirm the diagnosis.^[2]^ There is a classification system for JIA by the International League Against Rheumatism (ILAR) however, it doesn’*t* make an accurate diagnosis and hasn’*t* any specific biomarkers except for rheumatoid factor (RF), anti-cyclic citrullinated peptide (Anti-CCP) antibodies, and antinuclear antibody (ANA).^[3]^ So, a specific biomarker for JIA can prevent its diagnostic delay and complications, improving the outcomes.

About 1% of people worldwide suffer from rheumatoid arthritis (RA), a chronic inflammatory autoimmune illness with no known cause that strikes women two to three times more commonly than it does in males.^[4]^ Even though RA affects both small and big joints, symmetric extra-articular symptoms are frequently seen, particularly in cases where therapy is postponed. The eye may be impacted by necrotizing vasculitis of the small and medium-sized arteries, which in turn can also damage multiple organs.^[4]^ In rheumatoid arthritis, the eye is a common extra-articular source of inflammation. These conditions can range from benign ones like keratoconjunctivitis sicca and episcleritis to potentially vision-threatening ones like scleritis and peripheral ulcerative keratitis as well as potentially life-threatening problems. To effectively treat these individuals, ophthalmology and rheumatology should collaborate.^[5]^

All eukaryotic cells have internal chaperonins known as 14-3-3 Eta proteins. The human 14-3-3 isoforms are β, γ, ε, η, σ, τ/θ, and ζ (Beta, Gamma, Epsilon, Eta, Sigma, Tau/Theta, and Zeta), each having a unique name. They play a vital part in several intracellular biological processes, such as apoptosis, differentiation, and cell division.^[6,7]^

The 14-3-3 Eta protein functions as an inducer of the innate immune system when released into the extracellular space during the early phases of JIA and RA. Additionally, patients with arthritis have higher serum 14-3-3 Eta protein levels inducing the production of proinflammatory cytokines like tumor necrosis factor alpha (TNF-α), interleukin (IL)-6, and IL-1β, which can lead to joint degradation including matrix metalloproteinase 9 and receptor activator of nuclear factor-κB ligand (RANKL).^[6]^ Thus, 14-3-3 Eta protein may enhance laboratory effectiveness in the early diagnosis and prognosis of RA and JIA.^[8,9]^

The uveitis is one of the commonest and most dangerous extra-articular manifestations of JIA and RA especially in the oligoarthritic subtype presenting with chronic anterior uveitis with also non-specific biomarkers for its early detection.^[5,10]^ As 14-3-3 Eta protein is a proinflammatory cytokine inducer, it may therefore be a prospective and useful diagnostic tool for evaluating JIA and RA patients at risk of uveitis. Although the importance of 14-3-3 Eta proteins in several rheumatic diseases has been evaluated, its significance in the early identification of uveitis linked to JIA and RA is still being debate.^[2,11]^ In this study, serum 14-3-3 Eta protein was compared to other biomarkers, such as RF and Anti-CCP, to assess its potential as a novel biomarker for the early diagnosis of uveitis in Egyptian patients with JIA and RA, as well as its correlation with disease activity, was conducted.

## Patients and Methods

### Study Design and Patient Groups

Based on review of past literature^[6]^ who found that receiver operating curve (ROC) showed that serum14-3-3 Eta protein at a cutoff value of greater than 0.24 μg/L had a sensitivity of 88.89%, a specificity of 91.43%, a positive predictive value of 93%, and a negative predictive value of 86%, and area under curve (AUC) was 0.957, the least sample size calculated using statistics and sample size pro is 42 participants per each group. The power of study is 80% with 95% confidence interval.

This study’s case-control component comprised three patient groups: Group I involves 42 patients with JIA, divided into three subtypes including enthesitis, oligoarthritic, and polyarthritic JIA, recruited from the rheumatology clinic from September 2022 to May 2023 with an age of < 18 years who met the ILAR classification criteria for JIA.^[12]^ Group II includes 42 patients with RA diagnosed according to the 2010 American College of Rheumatology/European League against Rheumatism classification criteria for RA ^[13]^ in addition to an equal number of apparently healthy individuals for each group of patients matched in sex and age as a control group.

Exclusions from the current study included people on eye drugs, people with autoimmune diseases or other chronic illnesses, people with infectious or inflammatory diseases, people with endocrine abnormalities, people with neurological or psychiatric diseases in the past or present, and people with cancer.

### Ethical Guidelines

The University Hospital Committee approved this study, which was carried out in accordance with the Declaration of Helsinki and was assigned the Institutional Review Board number 32024PMRR10. Informed permission was given to each study participant.

### Methods

Age, sex, height, weight, and body mass index were recorded for each patient. A comprehensive medical history was also obtained, covering the onset, course, duration, current medication, and family history. Clinical and physical examinations were performed, observing for bone deformities, morning stiffness, tender joint count (TJC), swollen joint count (SJC), muscle atrophy, and extra-articular involvement regarding lymph nodes (cervical, axillary, and inguinal lymph nodes). The eyes were examined for any clinical evidence of uveitis, and the nails were examined for signs of psoriasis, vasculitis, skin redness, scars, or rashes.

The Juvenile Arthritis Disease Activity Scales (JADAS), measured four parameters including, the active joint count, the physician’s global assessment of disease activity, the parent’s evaluation of the child’s overall well-being, and erythrocyte sedimentation rate (ESR) were used to assess disease activity in JIA patients.^[14]^

The Childhood Health Assessment Questionnaire (CHAQ) was utilized to assess the patient’s functional capacity. The questionnaire, which includes a 10 cm pain visual analog scale (VAS) and a 10 cm parent global VAS, will be completed by the parent or child. The CHAQ will use 4-point ordinal scales as its response modalities (0 = no problem, 1 = considerable difficulty, 2 = much difficulty, and 3 = impossible to do).^[15]^

Disease activity score28 (DAS28) was used to assess the disease activity in RA patients including 28 joint counts.^[16]^

### Laboratory Investigations

Complete laboratory tests were performed, including the measurement of inflammatory markers (c-reactive protein (CRP) and ESR) using the Westergren technique, the production of a rheumatoid factor (RF) titer using the latex agglutination method (RF Direct Latex; EDALAB, France), where the presence of agglutination was interpreted as a positive for RF, and the measurement of serum 14-3-3 Eta using enzyme-linked immunosorbent assay (ELISA).

### Collection of Blood Samples

Six ml of venous blood were drawn from all subjects by clean venipuncture from the cubital vein and divided into 3 tubes; two ml of whole blood was collected in vacutainer tubes containing 3.8% Sodium Citrate additive for ESR, and four ml of whole blood was collected in plain vacutainer and allowed to clot at 37 °C for 30 min. Serum was separated by centrifugation at the speed of 300–700 ×*g* and used for assay of RF, CRP, and aliquot was stored at -20 °C for assay of serum Human 14-3-3 protein Eta (YWHAH).

Biochemical and Immune-assays ESR was estimated using the Westergren method, CRP was estimated with nephelometry assay, and seropositivity of RF was detected with latex agglutination. The level of serum Human 14-3-3 protein Eta (YWHAH) (catalog 201–12–5619) was estimated by sandwich-based ELISA kits (SunRed, China) according to manufacturer instructions. ESR, CRP, complete blood count (CBC), liver, and kidney function tests, Anti-nuclear antibody ANA, Anti-CCP), and Serum 14-3-3 Eta were assessed by ELISA.

### Eye Examination

A comprehensive ophthalmological examination was performed on all study participants, which included testing visual acuity using the Snellen Acuity Chart, measuring intraocular pressure with the Goldman Applanation Tonometer, examining the conjunctiva under a slit lamp for conjunctival hyperemia, checking the cornea for keratic precipitates, checking the anterior chamber for cells and flair, checking the iris and pupil for synechia, and checking the anterior vitreous to rule out posterior uveitis.

### Statistical Analysis

Using an IBM-compatible computer, SPSS (Statistical Package for the Social Sciences, Inc., Chicago, IL, USA) statistical package version 26 was used to tabulate and analyze the obtained data. For qualitative data, the expressions were numbers and percentages (No& %), whereas for quantitative data, they were mean, standard deviation (SD), and range. The study employed the Pearson Chi-squared test (χ^2^) to compare qualitative factors among the subgroups under investigation. If any of the predicted cells in the 2 × 2 table are less than five, the qualitative variables were compared using the Fisher exact test. The Mann-Whitney *U* test (*U*) is used to evaluate the difference between two quantitatively not regularly distributed data sets, whereas the student t-test is the test of significance used to examine the difference between two quantitatively normally distributed variables. The relationship between more than two quantitative, non-normally distributed variables was investigated using the Kruskal-Wallis’s test. The relationship between two quantitative, non-normally distributed variables was demonstrated using Spearman correlation. The test’s accuracy is measured by the area under the ROC curve (AUROC). A test with an area of 1 is considered perfect; one with an area of 0.5 is considered useless. The percentage of sick patients who test positive is known as sensitivity. The percentage of disease-free patients who test negative is known as specificity. The percentage of patients with positive tests who also have the disease is known as the predictive value of a positive test. The percentage of patients with negative test results who do not have the illness is known as the predictive value of a negative test. The threshold of statistical significance was established as a *P*-value < 0.05.

## Results

### Availability of the Data

The article incorporates the data used to substantiate the outcomes of this investigation.

A case-control study was conducted on 84 patients with JIA, and RA was divided into two groups equally, with 42 patients in each group.

Table 1 shows the demographic data among the two patient groups, there was a female predominance in both groups as there was 71.4% in the JIA group, and 85.7% in the RA group. The mean age in JIA group was 12.17 ± 3.36, versus 46.27 ± 6.44 in RA group, mean disease duration was 2.81 ± 0.86 in JIA group versus 7.12 ± 2.39 in RA group, the mean serum level of Eta protein was 29.52 ± 34.68 in JIA group versus 38.90 ± 33.41 in RA group, the mean of TJC, SJC, patient global assessment (PGA), and physical global assessment (PhGA) among JIA group were 4.67 ± 2.06, 2.79 ± 2.13, 5.43 ± 1.50, and 6.00 ± 1.53 respectively versus 2.88 ± 3.25, 1.46 ± 2.55, 5.02 ± 2.35, and 5.12 ± 1.82 in RA group, mean ESR, CRP, health assessment questionnaire (HAQ), was 40.12 ± 31.01, 13.05 ± 12.44, 1.57 ± 0.80 respectively, versus 28.73 ± 31.13, 11.02 ± 12.00, 1.73 ± 1.05 in RA group. The mean ANA was 1.97 ± 1.74 in JIA group versus3.38 ± 2.86 in RA group respectively with significant (*P* = 0.019, Table 1).

**Table 1:** Demographic data among the two patients’ groups.

Variable	JIA (*n* = 42)		RA (*n* = 42)		*P* value

	No.	%	No.	%	
Sex					
Male	12	28.6	6	14.3	0.111
Female	30	71.4	36	85.7	
Age (Years)	12.17 ± 3.36		46.27 ± 6.44		<0.001*
BMI (kg/m^2^)	17.21 ± 1.46		29.63 ± 3.09		<0.001*
Disease duration					
(Years)	2.81 ± 0.86		7.12 ± 2.39		<0.001*
Eta (μg/L)	29.52 ± 34.68		38.90 ± 33.41		0.027*
TJC	4.67 ± 2.06		2.88 ± 3.25		<0.001*
SJC	2.79 ± 2.13		1.46 ± 2.55		<0.001*
PGA	6.00 ± 1.53		5.02 ± 2.35		0.002*
PhGA	5.43 ± 1.50		5.12 ± 1.82		0.353
DAS28	----		3.69 ± 1.77		----
JDAS	16.25 ± 7.28		----		----
ESR	40.12 ± 31.01		28.73 ± 31.13		0.001*
CRP	13.05 ± 12.44		11.02 ± 12.00		0.029*
ANA	1.97 ± 1.74		3.38 ± 2.86		0.019*
HAQ	1.57 ± 0.80		1.73 ± 1.05		0.458
Severity					
Remission	0	0	16	38.1	<0.001*
Low	28	66.7	0	0	
Mild	0	0	10	23.8	
Moderate	6	14.3	6	14.3	
High	8	19	10	23.8	
RF					
Positive	23	54.8	33	78.6	0.021*
Negative	19	45.2	9	21.4	
Subtypes					
Polyarthritis	23	54.8	----	----	----
Oligo-arthritis	15	35.7			
Enthesitis	4	9.5			
Dry eye	11	26.2	15	35.7	0.345
Uveitis	8	19	4	9.4	0.212
Complications					
Present	1	2.4	3	7.1	0.616
Absent	41	97.6	39	92.9	

*, statistically significant; χ^2^, Chi-squared test; *t*, Student *t* test; *U*: Mann-Whitney *U* test; ANA, antinuclear antibody; FE, Fisher exact test; *n* = number; JIA, juvenile idiopathic arthritis; RA, rheumatoid arthritis; BMI, body mass index; TJC, tender joint count; SJC, swollen joint count; PGA, patient global assessment; PhGA, physical global assessment; DAS28, disease activity score; JADAS, juvenile arthritis disease activity scales; ESR, erythrocyte sedimentation rate; CRP, C-reactive protein; HAQ, health assessment questionnaire; RF, rheumatoid factor; NA, antinuclear antibody.

The mean of DAS28 was 3.69 ± 1.77 in the RA patients’ group, and the mean JADAS was 16.25 ± 7.28 in the JIA group. Among the JIA patients’ group, about 54.8% of them had polyarthritis, while about 35.7% and 9.5% had oligoarthritis, and enthesitis, 26.2% of them had dry eye versus 35.7% in the RA group, 19% had uveitis versus 9.4% in RA group, and the eye complications were found among 2.4 of them versus 7.1% in RA group. Among the JIA patients’ group, about 54.8% were RF positive, *versus* 78.6% in RA patients’ group (Table 1).

There was a significant statistical deference among JIA patients’ group and controls regarding the serum level of 14-33 Eta protein (*P* < 0.001), with also a statistically significant deference found among RA patients’ group versus controls regarding the serum level of 14-3-3 Eta protein (*P* < 0.001, Table 2).

**Table 2:** Comparison between cases and control regarding Eta level

Variable	Cases (*n* = 42)	Control (*n* = 42)	Test of significance	*P* value
JIA				
Eta (μg/L)				
Mean ± SD	29.52 ± 34.68	6.46 ± 5.04	*U* = 6.82	< 0.001
Range	8-130	1-23		
RA				
Eta (μg/L)				
Mean ± SD	38.36 ± 33.19	10.67 ± 2.87	*U* = 6.70	< 0.001*
Range	10-115	6-20		

*, statistically significant; *U*, Mann-Whitney *U* test; *n*, number; JIA, juvenile idiopathic arthritis; RA, rheumatoid arthritis.

There was a significant indirect relationship between age, and body mass index (BMI) with serum level of 14-3-3 Eta protein in the JIA patients’ group, while a significant direct relationship between serum level of 14-3-3 Eta protein and disease duration, TJC, SJC, PGA, PhGA, JADAS28, ESR, CRP, and HAQ was observed (Table 3).

**Table 3:** Correlation between Eta and other parameters among juvenile arthritis and rheumatoid arthritis studied patients

Parameter	JIA		RA	

	Eta (μg/L)		Eta (μg/L)	

	rho	*P*-value	rho	*P*-value
Age (Years)	-0.416	0.006*	0.089	0.576
BMI (kg/m^2^)	-0.409	0.007*	-0.118	0.458
Disease duration (Years)	0.068	0.668	0.021	0.897
TJC	0.618	<0.001*	0.773	<0.001*
SJC	0.701	<0.001*	0.766	<0.001*
PGA	0.658	<0.001*	0.853	<0.001*
PhGA	0.672	<0.001*	0.760	<0.001*
DAS28	----	----	0.842	<0.001*
JDAS	0.959	<0.001*	--	---
ESR	0.796	<0.001*	0.642	<0.001*
CRP	0.692	<0.001*	0.662	<0.001*
ANA	0.917	<0.001*	0.824	<0.001*
HAQ	0.787	<0.001*	0.806	<0.001*

*, statistically significant; rho, correlation coefficient; JIA, juvenile idiopathic arthritis; RA, rheumatoid arthritis; BMI, body mass index; TJC, tender joint count; SJC, swollen joint count; PGA, patient global assessment; PhGA, physical global assessment; DAS28, disease activity score; JADAS, juvenile arthritis disease activity scales; ESR, erythrocyte sedimentation rate; CRP, C-reactive protein; HAQ, health assessment questionnaire; ANA, anti-nuclear antibody.

There was a statistically significant positive correlation between 14-3-3 protein Eta and ANA in the JIA patient group (*P* < 0.001, Table 3).

A direct relationship between serum level 14-3-3 protein Eta and age, disease duration, TJC, SJC, PGA, PHGA, DAS28, ESR, CRP, and HAQ was observed in RA patients’ group with indirect relationship between it and BMI (Table 3).

The diagnostic accuracy of the 14-3-3 protein Eta biomarker in the prediction of RA disease occurrence in patients versus controls was at a cut-off point of 14.5 μg/L, AUC of 0.924, 95% CI = 0.868–0.980, while its diagnostic accuracy in JIA patients was at an 8.5 μg/L cut off point, and AUC of 0.930 (95% CI = 0.875–0.985, Table 4).

**Table 4:** Diagnostic accuracy of Eta in prediction of disease

Diagnostic accuracy	JIA	RA
AUC	0.930	0.924
*P* value	<0.001*	<0.001*
95% CI	0.875-0.985	0.868-0.980
Cut off point	8.5	14.5
Sensitivity	98%	83%
Specificity	81%	90%
Accuracy	89%	87%
PPV	84%	90%
NPV	97%	84%

*, statistically significant; AUC, area under curve; CI, confidence interval; PPV, positive predictive value; NPV, negative predictive value; JIA, juvenile idiopathic arthritis; RA, rheumatoid arthritis.

The diagnostic accuracy of 14-3-3 protein Eta in the prediction of uveitis in RA patients was a cut-off point of 57.5, with AUC = 0.992, 100% sensitivity, 97% specificity, 98% accuracy, 0.637–0.969 95% CI, 91% positive predictive value (PPV), and 100% negative predictive value (NPV), while its diagnostic accuracy at a cut-off point 12.75 in JIA patients, with low sensitivity 63%, specificity, 62%, and accuracy62% to differentiate between cases with or without uveitis, PPV was 28%, NPV was 88%, with a 0.530 AUC (Table 5).

**Figure 1:**
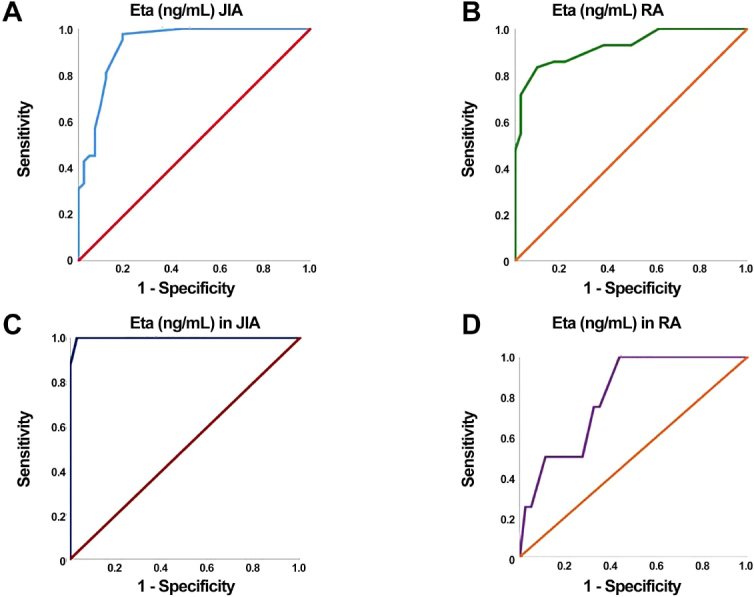
A, ROC curve for Eta as a predictor for JIA; B, ROC curve for Eta as a predictor for RA; C, ROC curve of Eta as a predictor of uveitis in JIA; D, ROC curve of Eta as a predictor of uveitis in RA. RA, rheumatoid arthritis; JIA, juvenile idiopathic arthritis.

**Table 5:** Diagnostic accuracy of Eta in prediction of uveitis

Diagnostic accuracy	JIA	RA
AUC	0.530	0.992
*P*-value	0.784	<0.001*
95% CI	0.282-0.777	0.972-1.000
Cut off point	12.75	57.5
Sensitivity	63%	100%
Specificity	62%	97%
Accuracy	62%	98%
PPV	28%	91%
NPV	88%	100%

*, statistically significant; AUC: area under curve; CI, confidence interval; PPV, positive predictive value; NPV, negative predictive value; JIA, juvenile idiopathic arthritis; RA, rheumatoid arthritis.

Analysis of each subtype of JIA showed elevated levels of 14-3-3 protein Eta in enthesitis, oligoarthritis and polyarthritis subtypes of JIA with the highest levels in the polyarthritis JIA group (17.25 ± 6.29, 13.70 ± 4.70, and 41.98 ± 43.14 respectively, Table 6).

**Table 6:** Subtypes of juvenile arthritis studied patients in relation to different clinical and laboratory parameters

Variable	JIA (*n* = 42)			*P* value

	Enthesitis (*n* = 4)	Oligoarthritis (*n* = 15)	Polyarthritis (*n* = 23)	
Eta (μg/L				
Mean ± SD	17.25 ± 6.29	13.70 ± 4.70	41.98 ± 43.14	0.020*
Range	10-23	9-25	8-130	
ESR				
Mean ± SD	26.25 ± 6.29	27.33 ± 12.66	50.87 ± 37.65	0.184
Range	20-35	10-50	10-125	
CRP				
Mean ± SD	5.75 ± 0.50	9.47 ± 5.19	16.65 ± 15.46	0.084
Range	5-6	4-24	4-48	
ANA				
Mean ± SD	1.25 ± 0.58	1.10 ± 0.70	2.67 ± 2.04	0.044*
Range	0.8-2.1	0.5-2.9	0.6-6.5	
Uveitis				
Present	0 (0 %)	0 (0 %)	8 (%34.8)	0.017
Absent	4 (100 %)	15 (100 %)	15 (65.2%)	

*, statistically significant; K, Kruskal Wallis test; χ^2^, Chi-squared test; JIA, juvenile idiopathic arthritis; ESR, erythrocyte sedimentation rate; CRP, C-reactive protein; ANA, anti-nuclear antibody.

There was statistically significant difference between enthesitis, oligoarthritis and polyarthritis subtypes of JIA and ANA with the highest level of ANA observed in the polyarthritis JIA group (*P* = 0.044, Table 6).

Regarding uveitis, no patients in the enthesitis or the oligoarthritis group had uveitis while eight patients in the polyarthritis group had uveitis (*P* = 0.017, Table 6).

## Discussion

Rheumatoid arthritis (RA) and juvenile idiopathic arthritis (JIA) are chronic inflammatory autoimmune illnesses with unclear causes**,** there is a considerable chance of major consequences, such as irreversible visual loss, if related uveitis develops in those patients. Consequently, it is crucial to screen at-risk patients for JIA, and RA-associated uveitis.^[17,18]^

In JIA patients, ANA is linked to uveitis, which if left undetected and untreated, could result in irreversible vision loss. To be clinically reliable, ANA testing is neither sensitive nor a specific predictor of uveitis risk, and it is unclear which characteristic autoantigens in JIA are specifically targeted by ANA. It is essential to discover particular autoantibodies that are highly correlated with uveitis since these can serve as biomarkers to help identify JIA patients who are at risk.^[19]^

One RA biomarker that is quite specific is the 14-3-3 Eta protein. In addition to providing a 15% additive advantage to the diagnostic sensitivity of the markers, RF, and CCP antibodies, it has predictive utility in identifying patients with early RA.^[9,20]^

Due to its correlation with the positivity of RF and anti-CCP in early JIA, previous studies have reported the role of serum 14-3-3 Eta protein in the early diagnosis of JIA especially the polyarthritis JIA group. Other studies have also shown the role of 14-3-3 Eta in determining the validity of the diagnosis of RA and the severity of early rheumatoid arthritis.^[9,18,21]^

Nevertheless, there is currently no proof estimating the effectiveness of the 14-3-3 Eta protein biomarker in JIA patients, either in identifying the disease’s activity or its role in identifying uveitis risk in JIA, and RA patients. Thus, in Egyptian JIA and RA patients, this is the first study to evaluate the relevance of blood 14-3-3 Eta protein as a possible novel biomarker for early uveitis identification.

The present study revealed the female predominance in JIA, and RA patients, as there were about 71.4%, and 85.7% of the studied patients among the two groups respectively, with seropositivity in about 54.8% of JIA patients, and 78.6% of RA patients. That agreed with previous studies that reported female predominance among 66.6%, 78%, and 85% respectively.^[21,22,23]^ Our findings reported a significant positive correlation between ANA and serum14-3-3 Eta protein in JIA patients, especially the polyarticular juvenile idiopathic arthritis (PJIA). Based on our search, there are few studies comparing the prevalence of positive ANA and serum 14-3-3 Eta protein. The study by Storwick *et al*., reported significant prevalence of ANA in JIA patients, stating that 30%–50% of JIA patients had positive ANA in varying percentages among the JIA subtypes which comes in agreement with our data.^[24]^

The study by Reyhan *et al*. comes in contrast to our findings reporting no association between positive ANA and 14-3-3 Eta. Overall, 60% of PJIA RF+ patients with positive ANA had serum 14-3-3 Eta levels above baseline. There was no association between positive ANA and 14-3-3 Eta among all groups (*P* = 0.119, OR = 1.96).^[21]^

According to the study by Risha *et al*.^[2]^, which included fifty JIA patients who met the ILAR classification criteria, there were statistically significant differences between the JIA and RA patient groups and the control group regarding the level of 14-3-3 Eta (μg/L)(*P* < 0.001). The study also found that JIA patients had significantly more serum 14-3-3 Eta proteins (27.7 μg/L) than healthy individuals (0.9 μg/L)(*P* < 0.05), and that these differences were positively correlated with RF and Anti-CCP especially in the polyarthritis JIA group.

Our findings are supported by Jianxin *et al*., which found that RA patients had considerably higher levels of 14-3-3 Eta expression. Receiver operating characteristic (ROC) analysis also suggested that this gene could be used as a predictive marker for RA risk. Serum levels of 14-3-3 Eta were positively linked in RA patients with erythrocyte sedimentation rate (*P* = 0.006), disease activity score in 28 joints (*P* = 0.025), and illness duration (*P* = 0.003).^[25]^

Also, the study by Abdelhafiz *et al*. comes in agreement with our findings estimating the role 14-3-3 Eta protein in RA which has now got adequate evidence for helping in assessing the veracity of the diagnosis and severity of early RA.^[9]^

Regarding the 14-3-3 Eta (μg/L) level, our study revealed statistically significant differences between JIA subtypes being detected in all subtypes of JIA group but most common and highest in the polyarthritis group. Several researches showed statistically significant differences between the groups of RA patients, JIA patients, and the control group.^[20,21,26,27]^

The current study reported a positive relationship between 14-3-3 Eta with clinical and laboratory indicators of disease activity, disease duration in JIA (*P* = 0.668), and RA (*P* = 0.897), CHAQ, and RF in both patients’ groups. Risha *et al*. documented the significant positive correlation between serum 14-3-3 Eta proteins in JIA patients and RF, Anti-CCP (*P* = 0.034 and 0.040, respectively), and CHAQ (*P* = 0.026) with no significant correlation with juvenile arthritis disease activity score 27 (JADAS27) score (*P* = 0.303).^[2]^ Serum levels of 14-3-3 Eta were shown to be favorably correlated with erythrocyte sedimentation rate (*P* = 0.006), disease activity score in 28 joints (*P* = 0.025), and illness duration (*P* = 0.003) in patients with RA, according to Jianxin *et al*.^[25]^

Also, several studies that documented the positive association between 14-3-3 Eta protein with laboratory markers of disease activity, and functional outcomes regarding CHAQ, reported the positive relationship between acute phase reactants (ESR & CRP), RF, clinical parameters regarding DAS28, number of swollen joints with 14-3-3 Eta level RA _patients._[2,9,23,26]

The 14-3-3 Eta level and RF had a statistically significant (*P* < 0.05) association. Whereby 14-3-3 Eta levels are greater in positive RF with respect to adult RA patients and JIA with the highest level in the polyarthritis group as compared to the enthesitis and oligoarthritis group. Compared to seronegative cases, the serum 14-3-3 Eta level was greater in adult RA (44.00 ± 35.31) and seropositive JIA (40.24 ± 43.17). These outcomes are comparable to those of JIA cases found by Risha *et al*.^[2]^

In contrast to our results, there was a study by Reyhan *et al*. and Risha *et al*. reported no significant association between age and disease activity using JADAS and 14-3-3 Eta level in JIA patients upon diagnosis.^[2,21]^

The diagnostic accuracy of the 14-3-3 Eta protein biomarker in prediction of RA disease occurrence in patients of the present study versus controls was at a cut-off point 14.5 μg/L, with AUC of 0.924, and 95% CI = 0.868–0.980, while its diagnostic accuracy in JIA patients was at an 8.5 μg/L cut off point, and AUC of 0.930 (95% CI = 0.875–0.985). The data from Wang *et al*. support our findings, which show that 14-3-3 Eta protein functions as a sensitive and specific biomarker for RA, with an area under the curve of 0.9245 and a pooled sensitivity and specificity of 0.73 (95% CI: 0.71–0.75) .^[28]^ In reference to JIA Risha *et al*. revealed that the serum 14-3-3 Eta protein RO) has been found to have 84% sensitivity, 55% specificity, 65.6% PPV, and 69.2% NPV among all JIA subgroup patients at levels greater than 1.45 μg/L ^[2]^

Numerous studies reported different cutoff points for 14-3-3 Eta protein among RA, JIA patients versus controls**,** this wide variations in the cut-off value in these studies may be related to the differences in ethnicity, disease duration, activity, and the biomarker reading range.^[27,29]^

Our study showed the prevalence of uveitis among the polyarticular subtype of JIA patients, being present in 8 (% 34.8) of 23 patients. The study by Qian *et al*., comes in agreement with our findings reporting the prevalence of uveitis in polyarticular JIA subtype.^[30]^ Also, the study by Reyhan *et al*. showed that 12 (33%) of the 36 polyarticular juvenile idiopathic arthritis (OJIA) patients had chronic uveitis which is consistent with our findings.^[21]^

In contrast to our study, the study by Heiligenh *et al*. which detected that uveitis is more common in the oligoarthritis subtype of JIA and rheumatoid factor–positive polyarthritis are rarely ( < 1%) accompanied by uveitis.^[31]^

The diagnostic accuracy of 14-3-3 Eta in prediction of uveitis in RA patients was at a cutoff point of 57.5, with 0.992 of AUC, 100% sensitivity, 97% specificity, 98% accuracy, 0.637–0.969 95% CI, 91% PPV, and 100% NPV, while its diagnostic accuracy at a cut-off point 12.75 in JIA patients, with low sensitivity 63%, specificity, 62%, and accuracy 62% to differentiate between cases with or without uveitis, PPV was 28%, NPV was 88%, with a 0.530 AUC.

Reyhan *et al*. study which shows that 12 (33%) of the 36 PJIA patients had chronic uveitis, is consistent with our findings. In polyarticular patients with 14-3-3 Eta (9/28; 33%), uveitis was as common as in those without 14-3-3 Eta (3/8; 38%), even though the numbers were too small for statistical analysis. Consistent with our findings, 14-3-3 Eta was not found in uveitis patients in other JIA groups.^[21]^

To the best of our knowledge, this study is the first to highlight and explore the role of 14-3-3 Eta protein biomarker in the early detection of uveitis among RA patients with a highly sensitive, specific, and accurate cutoff point, as, at 14-3-3 Eta protein biomarker serum level of 57.5, we could predict the occurrence of uveitis, and patients must be screened with a thorough ophthalmologic investigation at this cut-off point.

## Limitations of the Study

There are a few restrictions on the current study, one of which is the very small sample size. To prove the validity and dependability of our findings, more study with larger sample numbers is therefore necessary. Future research should hopefully concentrate more on the therapeutic effects of the sensitive biomarker 14-3-3 Eta protein than on its potential uses in diagnosis.

## Conclusion

14-3-3 Eta protein is a potential diagnostic biomarker in early detection of uveitis in RA patients, as it is higher in patients versus controls especially those with uveitis with a cut-off point 57.5, at which patients must have a thorough eye examination to receive early intervention and, to prevent complications, while it doesn’t have the same role in JIA patients. 14-3-3 Eta protein is a potential diagnostic and prognostic marker for JIA and RA being correlated with disease activity.
